# Right Hepatic Artery: A Cadaver Investigation and Its Clinical Significance

**DOI:** 10.1155/2015/412595

**Published:** 2015-12-16

**Authors:** Usha Dandekar, Kundankumar Dandekar, Sushama Chavan

**Affiliations:** ^1^Department of Anatomy, SMBT Institute of Medical Sciences and Research Centre, Dhamangaon, Nashik, Maharashtra 422403, India; ^2^Department of General Surgery, SMBT Institute of Medical Sciences and Research Centre, Dhamangaon, Nashik, Maharashtra 422403, India; ^3^Department of Anatomy, Rural Medical College, Pravara Institute of Medical Sciences, Deemed University, Loni, Rahata, Ahmednagar, Maharashtra 413736, India

## Abstract

The right hepatic artery is an end artery and contributes sole arterial supply to right lobe of the liver. Misinterpretation of normal anatomy and anatomical variations of the right hepatic artery contribute to the major intraoperative mishaps and complications in hepatobiliary surgery. The frequency of inadvertent or iatrogenic hepatobiliary vascular injury rises with the event of an aberrant anatomy. This descriptive study was carried out to document the normal anatomy and different variations of right hepatic artery to contribute to existing knowledge of right hepatic artery to improve surgical safety. This study conducted on 60 cadavers revealed aberrant replaced right hepatic artery in 18.3% and aberrant accessory right hepatic artery in 3.4%. Considering the course, the right hepatic artery ran outside Calot's triangle in 5% of cases and caterpillar hump right hepatic artery was seen in 13.3% of cases. The right hepatic artery (normal and aberrant) crossed anteriorly to the common hepatic duct in 8.3% and posteriorly to it in 71.6%. It has posterior relations with the common bile duct in 16.7% while in 3.4% it did not cross the common hepatic duct or common bile duct. The knowledge of such anomalies is important since their awareness will decrease morbidity and help to keep away from a number of surgical complications.

## 1. Introduction

One of the important structures closely involved in mishaps of hepatobiliary surgery is the right hepatic artery (RHA). The available laparoscopic, surgical, and imaging studies of RHA provide plenty of information but are limited to the field of vision or small field of surgery and cannot trace an anomalous artery, so dissection study of RHA was thought to correlate these findings in the hope of anatomical contribution to surgical safety.

The hepatic arteries provide 25% of blood supply and about 50% of oxygen supply to the liver [1]. The anatomy of hepatic artery is of great importance in hepatobiliary surgery, especially in cholecystectomy and liver transplantation. According to standard textbooks, the RHA usually arises from the proper hepatic artery (PHA) which is a continuation of common hepatic artery (CHA) usually to the left of the common hepatic duct (CHD). After its origin, the RHA runs upward and turns to the right, crossing behind the CHD to enter Calot's triangle. As it approaches the cystic duct (CD), it gives off the cystic artery (CA) and then turns upward to enter the right lobe of the liver. It almost always divides into an anterior branch supplying segments V and VIII and a posterior branch supplying segments VI and VII. The anterior division often supplies a branch to segment I and the gall bladder [2, 3]. When the RHA does not arise from the PHA or CHA, its origin is shifted to the aorta or any of the arteries whose normal course is towards right side of the aorta like superior mesenteric artery (SMA), gastroduodenal artery (GDA), right gastric artery, or celiac trunk (CT) [4, 5]. When the hepatic artery arises from a source other than the terminal end of the CT, it is considered as an aberrant hepatic, usually found in one-third of the cases. These aberrant hepatic arteries are of two types: replaced and accessory. A replaced hepatic artery is a substitute for the normal hepatic artery which is absent. An accessory hepatic artery appears in addition to one that is normally present [6, 7]. Michels stated that there are no accessory hepatic arteries since each hepatic artery is an end artery with a selective distribution to a definite area of the liver and therefore cannot be sacrificed without resultant necrosis of liver tissue [8]. The RHA occasionally forms a sinuous tortuosity called caterpillar hump or Moynihan's hump which occupies the major portion of Calot's triangle. It lies in close relation to the neck of the gall bladder or CD [3]. Injury to the RHA is more common in presence of aberrant arterial anatomy. These variations contribute to occurrence of potential problems during surgery leading to significant morbidity and even mortality. Adequate knowledge of normal and variant hepatic arterial anatomy is crucial for hepatobiliary surgery and liver transplantation. The aim of this cross-sectional, observational, quantitative, and descriptive study was to record the normal and variant anatomy of the RHA to contribute the existing knowledge of RHA to improve surgical safety.

## 2. Material and Methods

60 adult embalmed cadavers (males: 56; females: 4) with normal subhepatic anatomy were studied in the department of anatomy. Cadavers with operative procedure in subhepatic region or any subhepatic pathology like tumors were excluded. Specimens with topographical derangements were also excluded from this study. The dissection of subhepatic region was performed carefully to display the RHA and its related structures. Variation in the origin and course of the RHA as well as its relations with the hepatic ducts was recorded and appropriate photographs were taken.

## 3. Results

**Figure 1:**
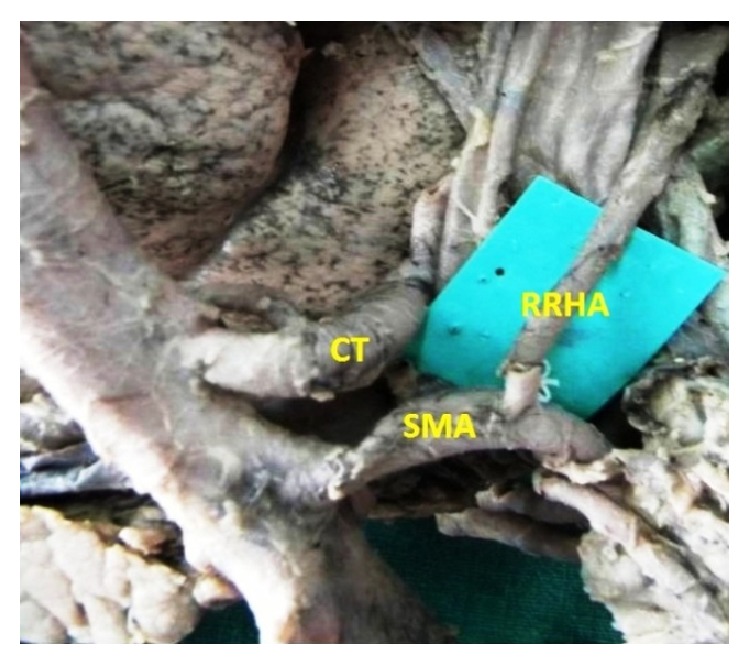
Replaced origin of RHA from SMA.

**Figure 2:**
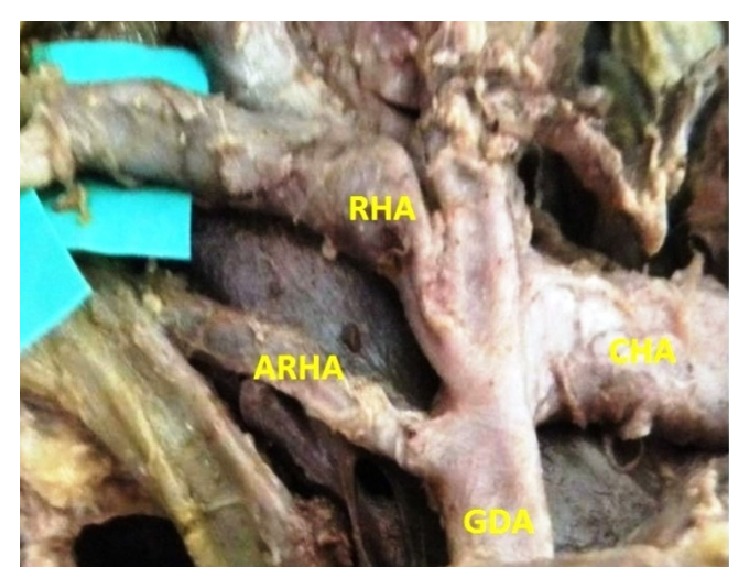
Accessory RHA arising from GDA.

**Figure 3:**
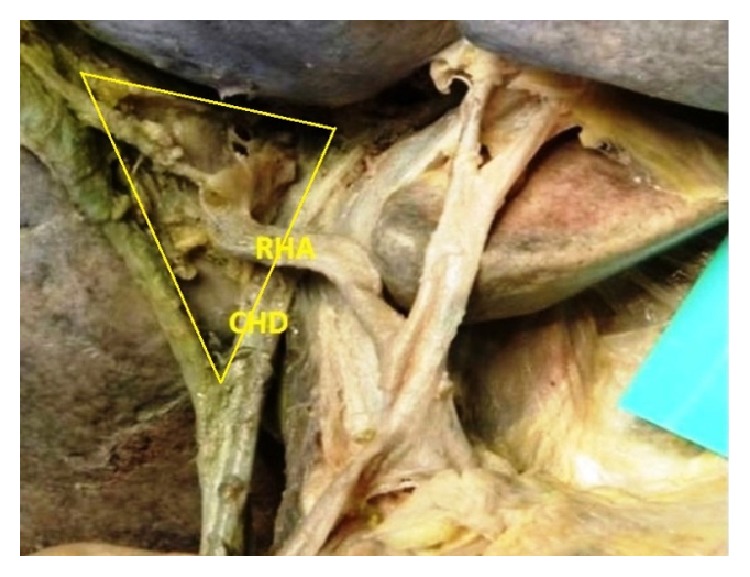
RHA crossing CHD anteriorly and entering Calot's triangle.

**Figure 4:**
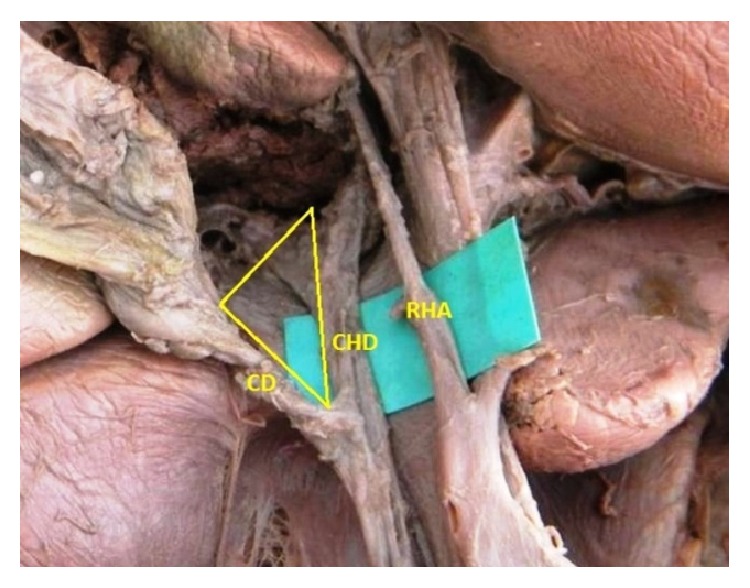
RHA coursing outside Calot's triangle.

**Figure 5:**
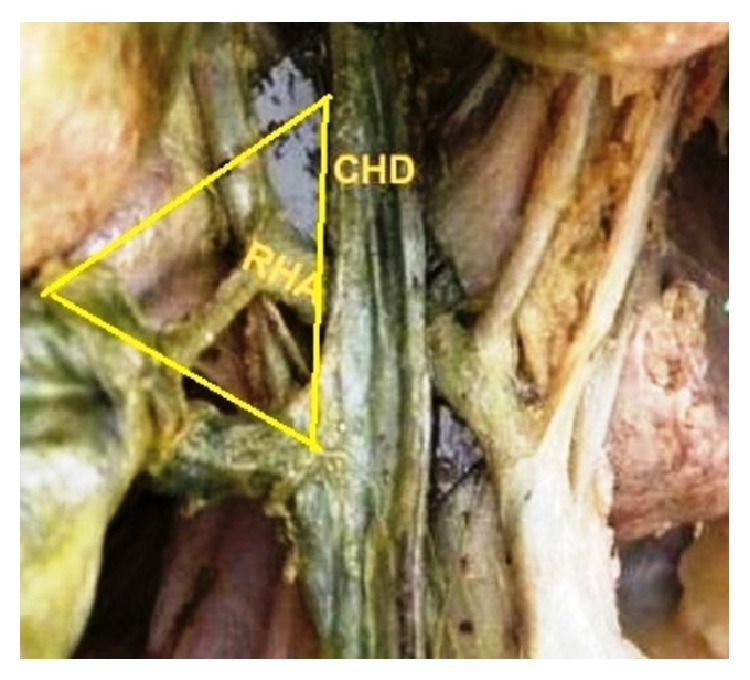
Course of RHA posterior to CHD.

**Figure 6:**
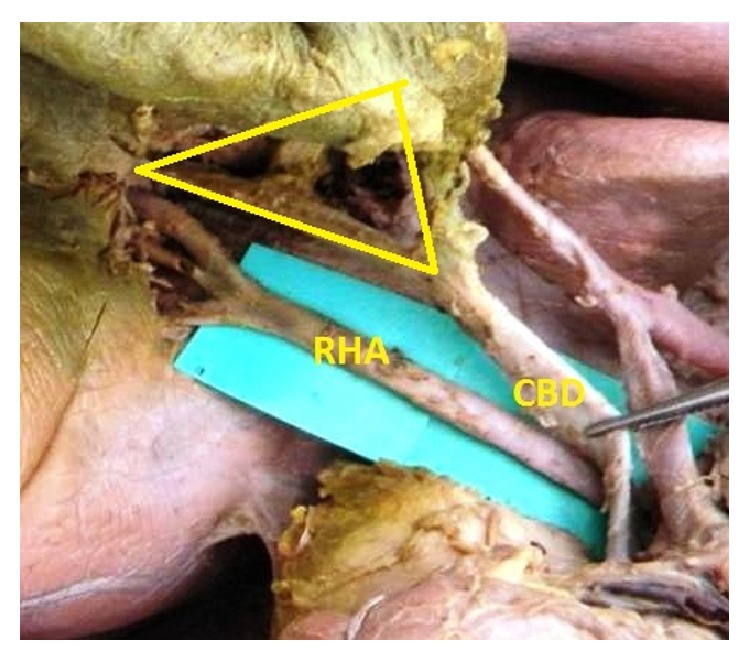
RHA coursing posterior to CBD.

**Figure 7:**
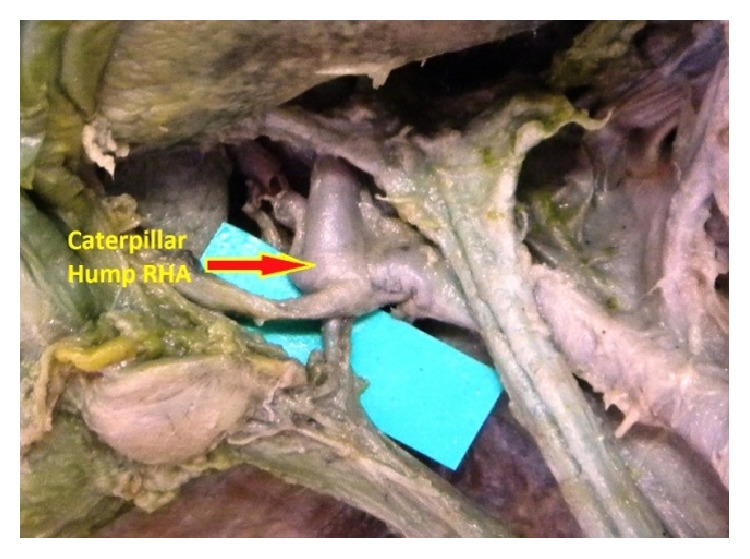
Caterpillar hump RHA.

The origin of the RHA from PHA or CHA was seen in 78.3% and, in the remaining 21.7% of the cases, it was aberrant. Replaced right hepatic arteries (RRHAs) were seen in 18.3% (Figure 1) while accessory right hepatic arteries (ARHAs) were seen in 3.4% of the cases (Figure 2). RRHA arose from SMA in 13.3%, from CT in 3.3%, and directly from aorta in 1.7% of the cases. ARHAs were seen to arise from GDA in 1.7% and from CHA in 1.7% of the cases. The RHA entered Calot's triangle in 95% (Figure 3) while in 5% it remained outside the triangle (Figure 4). The RHAs passing through the middle portion of Calot's triangle were observed in higher proportion (Table 1). Considering the relationship to the hepatic ducts, the RHA (normal and aberrant) crossed anteriorly to CHD in 8.3% (Figure 3) and posteriorly to it in 71.6% (Figure 5). It crossed common bile duct (CBD) posteriorly in 16.7% (Figure 6) whereas it had no relation with CHD or CBD in 3.4% of the cases. During its course, it gave off CA in 91.6% of the cases. Most of the RHAs coursed upwards to enter the right lobe of the liver, but few of them had very tortuous course, which made a “caterpillar hump.” In our study we found this hump in 8 specimens (13.3%) (Figure 7). Out of them, one loop in 2 specimens and 2 loops in 6 specimens were noted. In the latter, the CA arose from convexity of proximal loop in 2 specimens and from convexity of distal loop in 4 specimens.

## 4. Discussion

The “classic” hepatic arterial anatomy is present in approximately 55–75% of the cases [8, 19]. According to the literature, normal origin of RHA from PHA was seen in 80.4% of the cases (Table 2). The incidence of aberrant RHA arising from the SMA is higher as reported by various authors. Other sources are CT, aorta, GDA, or middle colic artery [4, 5, 9–13]. In the present study, similar finding, that is, origin of aberrant RHA from SMA, was found in higher proportion, that is, 13.3% of specimens. Other sources were CT (3.3%), aorta (1.7%), GDA (1.7%), and CHA (1.7%).

Michels's classic autopsy series of 200 dissections published in 1960 defined ten different types of anatomic variations of hepatic artery and has served as the benchmark for all subsequent contributions in this area [8]. López-Andújar et al. [20] investigated 1081 donor livers and compared the findings with Michels's classification. They found 2 new types which are not included in Michels's classification. Hiatt et al. [19] and Abdullah et al. [21] modified Michels's classification and classified the hepatic arteries into six types.

Bergman et al. [7] quoted the findings of Daseler et al. regarding the variable relations of the RHA with duct system in 500 specimens. In the study reported by Flint [9] in 200 cadavers and another study by Johnston and Anson [14], the various relationship of RHA with CHD and CBD was also mentioned. In the above literature, the posterior arterial relations were more common as compared to anterior arterial relations. The incidence of posterior arterial relations of CBD was less as compared to posterior arterial relations of CHD. In the present study, the findings are more or less similar to Johnston and Anson study (Table 3).

Caterpillar or Moynihan's hump RHA is a rare anomaly with insignificant appearance but has potential of creating catastrophe if injured. Devi quoted the incidence of caterpillar hump RHA in 5–15% of the cases as reported by Benson and Page [22]. Other authors like Flint [9], Johnston and Anson [14], Devi [22], and Mishal and Rajgopal [23] also reported it in 4%, 2.86%, 5%, and 1.6%, respectively. In the present study, it was much higher as 13.3%.

The tortuous artery may pass posteriorly or anteriorly to the CHD. The former is more common [24]. Devi reported that the caterpillar hump RHA passed posteriorly to CHD in 2 specimens and anteriorly to it in 1 specimen [22]. In the present study, it coursed behind the CHD in 4 specimens and in front of CHD in 2 specimens. The caterpillar hump may have single or double loops. The latter is commoner. In double looped hump, the CA can arise from either proximal or distal loop. Origin from the latter is more frequent. The CA, when arising from proximal loop, is long and crosses over the tortuous RHA to reach the gall bladder. If it arises from distal loop, it is very short owing to loop's proximity to the gall bladder [24]. In the study done by Devi, single loop was present in 1 specimen and 2 loops were present in 2 specimens. In the latter, the CA arose from the distal loop of caterpillar hump [22]. In the present study, we found single loop in 2 specimens and 2 loops in 6 specimens. The CA arose from convexity of proximal loop in 2 specimens and from distal loop in 4 specimens.

The RHA gives off CA in almost 90–95% of the cases [3]. Many authors have studied the incidence of origin of CA from RHA [6, 15–18]. Our findings correspond with the findings of Saidi et al. [15] and Khalil et al. [16] (Table 4).

The hepatic artery variations can usually be explained in terms of developmental basis. The liver is supplied during the fetal life by 3 arteries—right hepatic artery from SMA, left hepatic artery from left gastric artery, and common hepatic artery from the celiac trunk. With further development, the blood supply assumes the adult pattern, with atrophy of both right and left hepatic arteries, and the CHA gives off the right and left hepatic arteries supplying the whole liver. Anatomical variations correspond to the result of partial or complete persistence of the fetal pattern [5, 21].

## 5. Surgical Significance of Anomalous Right Hepatic Artery

The RHA variations and anomalies are not just anatomical study concerns but in reality play very crucial role in surgical mishaps. The RHA, as it crosses the bile ducts near the junction of the CD, is liable to injury during cholecystectomy [9]. The RHA may be mistaken for CA and liable to get ligated [24]. An aberrant RHA arising from the SMA or the aorta may run behind the portal vein [3]. In pancreatectomy and in operations on the duodenum, an aberrant RHA may be ligated compromising the blood supply to the right lobe. The accessory RHA may be injured during resection of the pancreatic head because the artery lies in close proximity to the portal vein [2]. Due to the variant course, the RHA comes in close proximity to CD and gall bladder. This results in formation of short CA; thus RHA may be mistaken for CA and inadvertently ligated during surgical procedures like cholecystectomy and liver transplantation [22, 25]. The “caterpillar hump” RHA is susceptible to iatrogenic injuries when bleeding obscures the field and it is the caterpillar loop that gets commonly injured while surgeon is jabbing and trying to control the hemorrhage. A “caterpillar hump” RHA can pass in front of or behind the CHD or CBD and may be mistaken for the CA and may get ligated [22]. The CA arising from “caterpillar hump” RHA is typically short and may get easily avulsed from the RHA, if excessive traction is applied to the gall bladder, producing serious hemorrhage [22, 23]. The presence of replaced RHA can be life-saving in patients with bile duct cancer because they are further away from the bile duct and tend to be spared from the cancer, making excision of the tumor feasible [2].

## 6. Conclusion

The RHA, being an end artery supplying right lobe of the liver, is important landmark in hepatobiliary surgery. It is to be meticulously sought and to be preserved as injury to it is known to cause necrosis of right lobe of the liver. It is essential from surgeon's viewpoint to have thorough knowledge and awareness of anomalies of RHA to prevent possible surgical complications.

## Figures and Tables

**Table 1:** Position of RHA within Calot's triangle.

Position within Calot's triangle	Number of specimens (95%)
Upper	33.3%
Middle	40%
Lower	21.7%

**Table 2:** Incidence of variations of origin of RHA.

Origin of RHA	Flint [9] %	Jones and Hardy [4] %	Bhardwaj [5] %	Ugurel et al. [10] %	Stauffer et al. [11] %	Sehgal et al. [12] %	Sureka et al. [13] %	Present study %
PHA/CHA	79	75	85	77	83.8	83.7	79.6	78.3

Replaced RHA								
SMA	21	18	8.3	19	12.1	11.6	13.5	13.3
CT	—	—	6.7	—	—	2.33	1.33	3.3
Aorta	—	1	—	1	—	—	0.33	1.7
MCA	—	—	—	1	—	—	—	—
GDA	—	6	—	—	—	2.33	—	—

Accessory RHA								
SMA	3.5	—	1.7	2	2.6	8	3.5	—
GDA	—	—	3.3	—	0.5	—	—	1.7
CHA	1	—	—	—	—	—	—	1.7
CT	—	—	—	—	—	6	1	—
Aorta	—	—	—	—	—	—	0.66	—

**Table 3:** Prevalence of variable relationship of RHA with CHD and CBD.

	Daseler quoted in [7] (%)	Flint [9] (%)	Johnston and Anson [14] (%)	Present study (%)
CHD				
Anterior	11.6	12.5	11.4	8.3
Posterior	65	68	74.3	71.6

CBD				
Anterior	1.4	—	—	—
Posterior	11.6	12.5	20	16.7

**Table 4:** Incidence of origin of cystic artery from RHA.

Michels [6] (%)	Saidi et al. [15] (%)	Khalil et al. [16] (%)	Bakheit [17] (%)	Pushpalatha and Shamasundar [18] (%)	Present study (%)
70	92.2	90	78	54	91.6

## Abbreviations

RHA: Right hepatic artery
PHA: Proper hepatic artery
CHA: Common hepatic artery
CHD: Common hepatic duct
CBD: Common bile duct
SMA: Superior mesenteric artery
GDA: Gastroduodenal artery
CT: Celiac trunk
CA: Cystic artery
CD: Cystic duct
RRHA: Replaced right hepatic artery
ARHA: Accessory right hepatic artery.
